# A serum-free adipose-conditioned medium delays stem cell senescence and maintains tissue homeostasis via IL-6/STAT3 axis suppression

**DOI:** 10.1186/s13287-025-04721-8

**Published:** 2025-11-28

**Authors:** Jing Li, Longzhu Song, Dongzi Song, Zheren Su, Jianhai Bi, Ran Huo

**Affiliations:** 1 Department of Burn and Plastic Surgery, Shandong Provincial Hospital Affiliated to Shandong First Medical University, Jinan, China; 2 Department of Burn and Plastic Surgery, Shandong Provincial Hospital, Cheeloo College of Medicine, Shandong University, Jinan, China; 3 Medical Science and Technology Innovation Center, Shandong First Medical University & Shandong Academy of Medical Sciences, Jinan, China

**Keywords:** Serum-free conditioned medium, Cellular senescence, Mesenchymal stem cells, IL-6/STAT3 signaling, Regenerative medicine

## Abstract

**Background:**

Stem cell exhaustion and cellular senescence are two hallmarks of aging. Mesenchymal stem cells (MSCs), as key players in tissue regeneration, are particularly vulnerable to senescence, which compromises both their endogenous regenerative capacity and their therapeutic efficacy in cell-based applications. Suppressing MSC senescence is therefore essential for developing effective regenerative and anti-aging strategies.

**Methods:**

We developed a serum-free adipose-conditioned medium (SF-ACM) from in vitro–cultured human adipose explants. Its anti-aging effects were evaluated in oxidative stress–induced and replicative senescence models of human adipose-derived stem cells (ADSCs), assessing proliferation, senescence markers, migration, and trilineage differentiation. Parallel experiments in senescent human dermal fibroblasts (HDFs) examined proliferation, fibrosis, and senescence features. In vivo, male C57BL/6 J mice (4 or 16 months old) with D-galactose–induced and naturally aged mice received intraperitoneal SF-ACM. Aging phenotypes were analyzed in skin, adipose tissue, muscle, kidney, and serum, along with hepatic and renal safety assessments.

**Results:**

SF-ACM significantly reduced senescence-associated markers, including p16, p21, p53, and SA-β-gal, enhanced Lamin B1 expression, and improved the proliferation, migration, and differentiation capacities of ADSCs. It also decreased senescence and fibrosis-related markers in HDFs. In aging mice, SF-ACM improved aging-associated phenotypes in skin, adipose tissue, and skeletal muscle. Mechanistically, these effects were associated with suppression of the IL-6/STAT3 signaling pathway.

**Conclusions:**

This study identifies a novel xenogeneic-free, paracrine-rich formulation that delays cellular senescence and preserves tissue homeostasis. These findings support its potential as a safe and effective strategy to suppress cellular aging, restore stem cell function, and ameliorate tissue-level aging, offering translational promise for regenerative medicine and anti-aging interventions.

**Supplementary Information:**

The online version contains supplementary material available at 10.1186/s13287-025-04721-8.

## Background

Aging is a fundamental biological process that impairs tissue regeneration and increases susceptibility to chronic diseases, posing a major challenge to regenerative medicine [1]. A key hallmark of aging is the accumulation of senescent cells—cells that undergo irreversible growth arrest and secrete pro-inflammatory factors collectively termed the senescence-associated secretory phenotype (SASP) [2, 3]. SASP factors disrupt tissue structure and function, contributing to fibrosis, impaired wound healing, and organ failure [4]. As a result, therapeutic strategies targeting senescence are gaining attention. These include senolytics, which eliminate senescent cells (e.g., dasatinib, quercetin), and senomorphics, which suppress SASP-driven inflammation (e.g., rapamycin, JAK inhibitors) [5, 6]. However, their clinical application remains limited by issues such as off-target effects, poor tissue specificity, and uncertain long-term safety [7].

Stem cell exhaustion is another hallmark of aging that compromises tissue repair capacity [2]. As adult stem cells lose their proliferative and differentiation potential, tissues exhibit diminished regenerative responses [8]. Although interventions such as caloric restriction, epigenetic reprogramming, and niche modulation show promise, most lack tissue specificity or carry systemic risks [9].

Mesenchymal stem cells (MSCs), especially adipose-derived stem cells (ADSCs), are widely used in regenerative medicine due to their multipotency and paracrine activity [10, 11]. However, ADSC function is sensitive to both donor-intrinsic factors (e.g., age, tissue origin) and extrinsic stressors (e.g., oxygen exposure, cryopreservation) [12]. Notably, ambient oxygen (~ 21%) induces oxidative stress that exceeds physiological levels in adipose tissue (3–6%) and accelerates senescence [13, 14]. As a representative MSC subtype, ADSCs display aging-associated features such as DNA damage, mitochondrial dysfunction, and SASP activation [4, 8]. Preserving ADSC function and delaying stress-induced senescence may therefore enhance their therapeutic potential [9, 15].

Explant culture, a method for primary cell isolation that involves culturing intact tissue fragments without enzymatic digestion, preserves native microarchitecture and paracrine signaling, and yields cells with greater phenotypic stability than enzymatic digestion methods [16, 17]. This raises the possibility that conditioned medium derived from explant culture may contain regenerative or anti-senescent factors. However, whether explant-derived media, particularly under serum-free conditions, can serve as a therapeutic source of such factors remains unknown.

To test this hypothesis, we collected both serum-containing (ACM) and serum-free (SF-ACM) adipose-conditioned media from cultured human adipose tissue explants under respective conditions. In oxidative stress–induced and replicative senescence models of ADSCs, both media alleviated senescence phenotypes, and SF-ACM was selected for further investigation due to its xenogeneic-free composition and translational potential.

SF-ACM restored ADSC proliferation, reduced senescence markers (e.g., p53, p21), and suppressed SASP factors (e.g., IL-8, TNF-α). It also promoted cell migration and multilineage differentiation. Similar anti-senescent effects were observed in human dermal fibroblasts (HDFs), including reduced senescence and fibrosis, and in aging mice, where SF-ACM improved multiple tissue aging phenotypes. Although transcriptomic changes varied between senescence models, IL-6 downregulation emerged as a shared target of SF-ACM. Follow-up experiments confirmed that SF-ACM suppressed IL-6/STAT3 signaling, identifying this axis as a central mechanism underlying its anti-senescent activity.

Interestingly, no cells migrated from adipose explants during the 14-day serum-free culture period, yet serum addition triggered rapid cell emergence within 2–3 days—much earlier than the 7–14 days typically required in standard explant protocols [18]. This suggests that adipose tissue remains metabolically active under serum-free conditions and continuously releases regenerative signals.

In this study, we report for the first time the development of a serum-free, xenogeneic- and cell-free platform using activated human adipose explants to harvest endogenous anti-aging factors. In both in vitro and in vivo models, SF-ACM attenuated stem cell senescence and improved tissue phenotypes, suggesting its potential as a therapeutic approach to mitigate stem cell aging and age-related tissue dysfunction.

## Methods

### Patients and samples

Adipose tissues were collected from microtia patients undergoing auricular reconstruction, with redundant chest subcutaneous fat harvested during costal cartilage collection. Human skin samples were obtained from patients undergoing reduction mammaplasty. All procedures were performed at the Department of Burn and Plastic Surgery, Shandong Provincial Hospital.

### Animal models

The work has been reported in line with the ARRIVE 2.0 guidelines. Male C57BL/6 J mice (4 or 16 months old; Vital River, Beijing, China) were housed under SPF conditions with a 12 h light/dark cycle and free access to food and water. Mice were acclimatized for 1 week before the experiment.

For the D-galactose–induced aging model, 4-month-old mice were randomly assigned (using a random number table) to four groups (n = 6 per group) and treated for 8 weeks as follows: (1–2) Mice in the control groups received intraperitoneal injections of 100 μL per injection, once weekly, of either normal saline or low-glucose DMEM (C11885500BT; Gibco, Thermo Fisher Scientific, Waltham, MA, USA); (3–4) Mice in the D-galactose groups received the same weekly intraperitoneal injections (100 μL of low-glucose DMEM or SF-ACM), and additionally received daily subcutaneous injections of D-galactose (HY-N0210; MedChemExpress, Monmouth Junction, NJ, USA; 100 mg/kg/day, 100 μL per injection). For the natural aging model, 16-month-old mice were randomly allocated into two groups (n = 6 per group) and received intraperitoneal injections of 100 μL per injection, once weekly, for 4 weeks: low-glucose DMEM or SF-ACM.

The experimental unit for all in vivo assays was a single mouse, resulting in a total of 36 animals across both models. Sample size (n = 6 per group) was selected based on previous studies using comparable models and with consideration of the 3R principles to minimize animal use [19, 20]. The primary outcomes for sample size consideration were histological parameters and molecular markers of aging. All animals were included at the start of the study. Pre-specified exclusion criteria were technical failure (e.g., insufficient sample yield, tissue damage during processing); otherwise, no exclusions were made. For each experiment, the exact n values are indicated in the figure legends.

Treatment order was randomized, and after each injection, mice were randomly relocated in the cage racks to minimize potential confounders. At the experimental endpoint, mice were euthanized by cervical dislocation performed by trained personnel, in accordance with the AVMA 2020 Guidelines for the Euthanasia of Animals. No anesthesia was used prior to euthanasia to avoid its effects on blood parameters and to ensure sufficient volumes of cardiac blood could be collected. Immediately after euthanasia, blood was collected by cardiac puncture, and tissues were harvested for histological analysis or snap-frozen at – 80 °C for molecular assays. Investigators performing histological and molecular analyses were blinded to group allocation, whereas treatment administration was performed by a separate investigator aware of group identity.

### Preparation of ACM and SF-ACM

Fresh adipose tissue was washed with sterile phosphate-buffered saline (PBS; PB180327; Pricella, Wuhan, China), dissected to remove fibrous tissue, and cut into cubes measuring 0.2 cm × 0.2 cm × 0.2 cm. Explants were evenly spaced (~ 0.2 cm) in culture dishes. To prepare ACM, explants were cultured in low-glucose DMEM supplemented with 10% fetal bovine serum (FBS; S660JJ; BasalMedia, Shanghai, China) for 6 days; conditioned medium was collected every 48 h before visible cell outgrowth. To prepare SF-ACM, explants were cultured in low-glucose DMEM under the same conditions for 14 days, and medium was collected every 48 h. All samples were centrifuged (2000×g, 10 min), filtered (0.22 μm), and stored at − 80 °C.

### Isolation of ADSCs and HDFs, and induction of senescence

ADSCs were isolated by digestion with 0.075% collagenase I (C0130; Sigma-Aldrich, St. Louis, MO, USA) for 30–60 min at 37 °C, followed by neutralization with complete medium, filtration through a 100 μm strainer, centrifugation at 190 × g, and culture under 3% O_2_. HDFs were obtained by removing the epidermis, mincing the dermis, and digesting with 0.1% collagenase I. Replicative senescence was induced by continuous passaging of ADSCs and HDFs under physiological oxygen (3% O_2_), while oxidative stress–induced senescence was modeled by culturing cells from passage 1 (P1) under normoxic conditions (21% O_2_).

### Treatment conditions

To evaluate dose responses, ADSCs were treated with 5%, 10%, 25%, or 50% (v/v) ACM or SF-ACM in DMEM supplemented with 10% FBS and 1% penicillin–streptomycin; total volume was normalized to 100%.

For pathway inhibition, recombinant human IL-6 (10 ng/mL; HY-P7044; MedChemExpress) and LMT-28 (30 μM; HY-102084; MedChemExpress), a selective IL-6/STAT3 inhibitor, were added.

Unless otherwise specified, P5 (21% O_2_) and P17 (3% O_2_) ADSCs and HDFs were treated for two passages, and the resulting P6 (21% O_2_) and P18 (3% O_2_) cells were used in functional assays.

### Flow cytometric characterization of ADSCs

P6 ADSCs (3% O_2_) were harvested at ~ 70% confluence and stained using the BD Stemflow™ Human MSC Analysis Kit (562245; BD Biosciences, San Jose, CA, USA), per manufacturer’s instructions. Samples were acquired on a Cytek Aurora full-spectrum flow cytometer (Cytek Biosciences, Fremont, CA, USA) and analyzed with FlowJo v10.8.1 (BD Biosciences, Ashland, OR, USA). Gates were set based on isotype controls.

### Senescence-associated β-galactosidase (SA-β-gal) staining

SA-β-gal staining was performed using a β-Galactosidase Staining Kit (G1580; Solarbio, Beijing, China) according to the manufacturer’s instructions. After staining at 37 °C (without CO_2_) for 24–48 h, cells were imaged using an Olympus CKX41 microscope (Olympus, Tokyo, Japan) with OPLENIC_CAMERA. The percentage of SA-β-gal–positive cells was calculated from five random fields per sample using Fiji (ImageJ, NIH, Bethesda, MD, USA).

### Population doubling level (PDL) assay

ADSCs were seeded at equal initial densities (N_0_) and allowed to attach for 12 h before treatment. When one group reached ~ 70% confluence, all groups were harvested, and total cell number (N_1_) recorded. Cells were then re-seeded at N_0_ to repeat the process. PDL was calculated using the formula log_2_(N_1_/N_0_).

### CCK-8 assay

ADSCs were incubated in DMEM containing 10% CCK-8 reagent for 2 h (E-CK-A362; Elabscience, Wuhan, China), and absorbance at 450 nm was measured at 0, 24, 48, and 72 h using a MULTISKAN FC microplate reader (Thermo Scientific). Data were normalized to 0-h absorbance for each group.

### Scratch wound assay

A linear scratch was made on fully confluent ADSCs using a sterile 200-μL pipette tip. Wound areas were imaged at 0 and 24 h using an Olympus CKX41 microscope. Wound closure was analyzed using Fiji software.

### Cell cycle analysis

Treated ADSCs were fixed in 70% cold ethanol overnight at 4 °C, then stained with PI/RNase staining solution (CY001-L; SIMUBIOTECH, Wuhan, China) for 30 min at room temperature in the dark. Cell cycle profiles were acquired using an Attune NxT Acoustic Focusing Cytometer (Thermo Fisher Scientific) and analyzed with FlowJo v10.8.1.

### Trilineage differentiation assays

For adipogenesis, ADSCs were induced for 2–3 weeks using the OriCell Adipogenic Kit (HUXMD-90041; Cyagen, Guangzhou, China), stained with Oil Red O (Sigma-Aldrich, St. Louis, MO, USA), and quantified from ≥ 5 random fields. For osteogenesis, ADSCs were cultured in Human MSC Osteogenic Medium (PD-007; Pricella) for 4 weeks, then stained with Alizarin Red S. For chondrogenesis, 3–4 × 10^5^ ADSCs were aggregated in 5-mL tubes and induced using the OriCell Chondrogenic Kit (HUXMD-90041; Cyagen) for 2 weeks. Pellets were fixed, paraffin-embedded, sectioned (5 μm), and stained with Alcian Blue. Pellet diameters were measured before embedding.

### Quantitative PCR (qPCR)

Total RNA was isolated using the FastPure Cell/Tissue RNA Isolation Kit V2 (RC112-01; Vazyme, Nanjing, China). First-strand cDNA was synthesized from 1 μg RNA using the SPARKscript II RT Plus Kit (AG0304-B; SparkJade, Shandong, China). qPCR was performed using 2 × SYBR qPCR Mix (AH0104-B; SparkJade) on a LightCycler^®^ 480 II system (Roche, Basel, Switzerland). Relative expression levels were calculated by the 2^–ΔΔCq method, using *ACTB* as the reference gene. Primer sequences were as follows: *ACTB* forward 5′-AGCACTGTGTTGGCGTACAG-3′, reverse 5′-AGAGCTACGAGCTGCCTGAC-3′; *IL6* forward 5′-ACTCACCTCTTCAGAACGAATTG-3′, reverse 5′-CCATCTTTGGAAGGTTCAGGTTG-3′.

### Western blotting

Cells were lysed in RIPA buffer with protease and phosphatase inhibitors. Protein (20 μg/lane) was resolved by SDS-PAGE, transferred to PVDF membranes (Millipore, Burlington, MA, USA), and blocked with QuickBlock™ (P0252; Beyotime, Shanghai, China). Membranes were incubated overnight at 4 °C with primary antibodies against p21 (2947S; Cell Signaling Technology; Danvers, MA, USA; 1:1000), p16 (80772S; Cell Signaling Technology; Danvers, MA, USA; 1:1000), p53 (10442-1-AP; Proteintech, Wuhan, China; 1:5000) and Lamin B1 (12987-1-AP; Proteintech, 1:5000), p-STAT3 (AP0705; Abclonal, Wuhan, China; 1:1000) and STAT3 (A19566; Abclonal, 1:1000), and β-actin (AB0035; Abways, Shanghai, China; 1:10000), followed by HRP-conjugated secondary antibody (AS014; Abclonal, 1:5000). Signals were detected with ECL (RM02867; Abclonal) and imaged using a ChemiDoc™ system (Bio-Rad, Hercules, CA, USA). Band intensities were quantified with Fiji and normalized to β-actin.

### Biochemical and cytokine assays

ADSC supernatants were collected 48 h after treatment in low-glucose DMEM. IL-6, IL-8, and IL-1β levels were measured using ELISA kits (RK00004, RK00011, RK00001; Abclonal). Absorbance was recorded at 450 nm (reference 570 nm) using a MULTISKAN FC reader (Thermo Scientific).

Plasma from treated mice was analyzed for IL-6 and IL-1β using ELISA kits (RK00008, RK00006; Abclonal). Serum urea nitrogen (BUN), alanine aminotransferase (ALT), and aspartate aminotransferase (AST) levels were assessed using colorimetric kits (E-BC-K183-M, E-BC-K235-M, E-BC-K236-M; Elabscience), and concentrations were calculated from standard curves.

### RNA sequencing and bioinformatics analysis

Total RNA was extracted from ADSCs using RNA isolator (R401-01; Vazyme) and dissolved in 0.1% DEPC-treated water. RNA quality was assessed using a NanoDrop spectrophotometer (Thermo Fisher Scientific) and a 5300 Bioanalyzer (Agilent Technologies, Santa Clara, CA, USA). Samples with RQN ≥ 6.5, 28S:18S ≥ 1.0, and yield ≥ 1 μg were sequenced (paired-end 150 bp) on the Illumina NovaSeq X Plus platform by Shanghai Majorbio Bio-pharm Biotechnology Co., Ltd.

Clean reads were filtered with fastp, aligned to the GRCh38 genome using HISAT2, and assembled with StringTie. Gene expression was quantified with RSEM and normalized as TPM. Differential expression was determined using DESeq2 (|log_2_FC|≥ 1, FDR < 0.05). Functional enrichment (GO and KEGG) was performed using Goatools and scipy with Bonferroni correction (P < 0.05). Alternative splicing was analyzed with rMATS, and data visualization was conducted on the Majorbio Cloud Platform (https://cloud.majorbio.com).

### Immunofluorescence, histology, and immunohistochemistry

ADSCs and HDFs were seeded on coverslips, fixed (4% paraformaldehyde), permeabilized (0.5% Triton X-100), and blocked (normal goat serum). Primary antibodies included p16 (A11651; Abclonal; 1:100), Ki-67 (HA601053; HUABIO, Hangzhou, China; 1:200), γ-H2AX (9718 T; CST, 1:200), α-SMA (GB13044; Servicebio, Wuhan, China; 1:200), and vimentin (10366-1-AP; Proteintech, 1:200). DyLight 488-conjugated secondary antibody (A23220; Abbkine, Wuhan, China; 1:200) and DAPI (C0065; Solarbio) were used for detection. Imaging was performed with an EVOS M7000 microscope (Thermo Fisher).

Paraffin-embedded mouse skin sections underwent standard deparaffinization, antigen retrieval, and staining with Lamin B1 (12987-1-AP; Proteintech; 1:100) and TGF-β1 (CPA2154; Cohesion Biosciences, London, UK; 1:200), followed by secondary antibody and DAPI. Lamin B1 images were captured on a Zeiss LSM 900 confocal microscope.

Mouse skin, kidney, and muscle were fixed (4% paraformaldehyde or GD muscle fixative; G1111; Servicebio), paraffin-embedded, and sectioned (5 μm). H&E (G1080, G1100; Solarbio), Masson’s trichrome (G1340; Solarbio), and Sirius Red (G1472; Solarbio) were used to assess morphology, collagen, and fibrosis. Images were acquired with an Olympus CX31 (Olympus). Fiji was used to quantify epidermal/dermal thickness, hair follicle counts, muscle fiber area, glomeruli, and interstitial fibrosis.

Frozen adipose section (10 μm) were fixed, permeabilized (0.3% Triton X-100), blocked (5% BSA), and stained with p53 (10442-1-AP; Proteintech; 1:500) and p21 (GB115313; Servicebio; 1:250), followed by HRP-conjugated secondary antibody and DAB. Hematoxylin counterstaining was performed. Images were acquired using an Olympus CX31, and DAB-positive nuclei were quantified using Fiji.

### Statistical analysis

All data exclusion criteria were prospectively defined. Data were only excluded in cases of technical failure (e.g., sample damage, incomplete processing, or assay failure), but no such exclusions occurred in this study. Statistical analyses were conducted in GraphPad Prism 9.0. Data are shown as mean ± SD. Normality was tested with the Shapiro–Wilk test. Student’s t test or ANOVA with Tukey’s post hoc test was used as appropriate. P < 0.05 was considered significant. All experiments included ≥ 3 biological replicates.

## Results

### ADSC phenotype and oxygen-dependent senescence models

Primary human ADSCs were isolated and expanded in vitro. Flow cytometry confirmed their mesenchymal phenotype (> 99% CD105⁺, CD90⁺, CD73⁺; CD45⁻, CD11b⁻, CD19⁻, HLA-DR⁻; Supplementary Figure S1), consistent with MSC criteria [21].

To model distinct aging processes, we cultured ADSCs under 3% (physiological) or 21% (normoxic) oxygen, based on evidence that normoxia accelerates oxidative stress–induced senescence while low oxygen maintains replicative capacity [13, 14, 22].

**Fig. 1:**
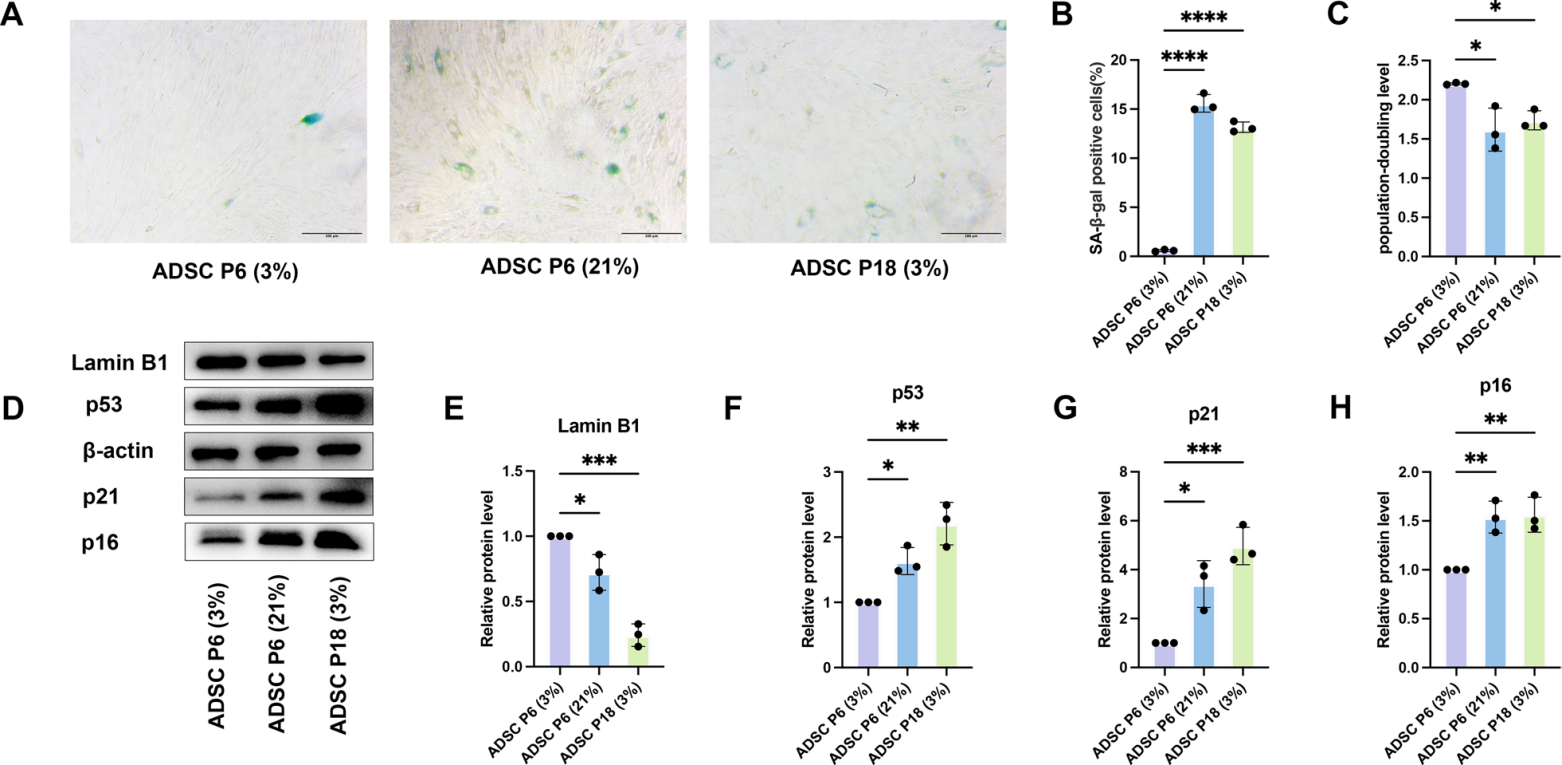
Oxygen tension determines distinct senescence phenotypes in ADSCs. **A** Representative images of SA-β-gal staining in ADSC P6 (3%), ADSC P6 (21%), and ADSC P18 (3%). Scale bar, 100 μm. **B** Quantification of the percentage of SA-β-gal–positive cells in each group. **C** PDL of ADSCs at early and late passages under 3% or 21% O_2_. **D** Western blot analysis of senescence markers (p53, p21, p16, and Lamin B1) in ADSC P6 (21%) and ADSC P18 (3%); ADSC P6 (3%) served as control. β-actin served as the internal loading control. **E**–**H** Densitometric quantification of protein expression normalized to β-actin and expressed relative to ADSC P6 (3%) (set as 1.0). All data are presented as mean ± SD from three independent experiments. One-way ANOVA followed by Tukey’s post hoc test was used for statistical analysis; *P < 0.05, **P < 0.01, ***P < 0.001, ****P < 0.0001

Compared with P6 ADSCs at 3% O_2_ [ADSC P6 (3%)], both P6 ADSCs at 21% O_2_ [ADSC P6 (21%)] and P18 ADSCs at 3% O_2_ [ADSC P18 (3%)] showed typical senescence features, including increased SA-β-gal staining (Fig. 1A–B), reduced PDL (Fig. 1C), upregulated p53, p21, and p16, and downregulated Lamin B1 (Fig. 1D–H). These markers reflect irreversible growth arrest and nuclear structural decline.

Notably, SA-β-gal⁺ cells were rare in ADSC P6 (3%) (0.6% ± 0.3%) but significantly elevated in ADSC P6 (21%) (15.6% ± 0.5%), underscoring the role of oxygen tension in modulating ADSC senescence.

### SF-ACM prevents but does not reverse senescence in ADSCs

During conventional explant-based isolation of primary cells, the culture medium typically contains ≥ 10% FBS [16]. To evaluate the anti-senescence potential of ACM while addressing the well-known limitations of FBS—including undefined composition, batch variability, and xenogeneic risks—we prepared both ACM and a parallel serum-free version (SF-ACM) [23, 24]. In FBS-containing cultures, ADSCs typically began migrating from adipose tissue explants around days 6–7. In contrast, when adipose tissue was maintained under serum-free conditions for 14 days, no outgrowth of ADSCs was observed. Notably, upon reintroduction of FBS after serum-free incubation, cell migration resumed within 2–3 days—markedly faster than in standard explant-based isolation—indicating that the tissue remained viable and paracrine-active throughout the serum-free period.

**Fig. 2:**
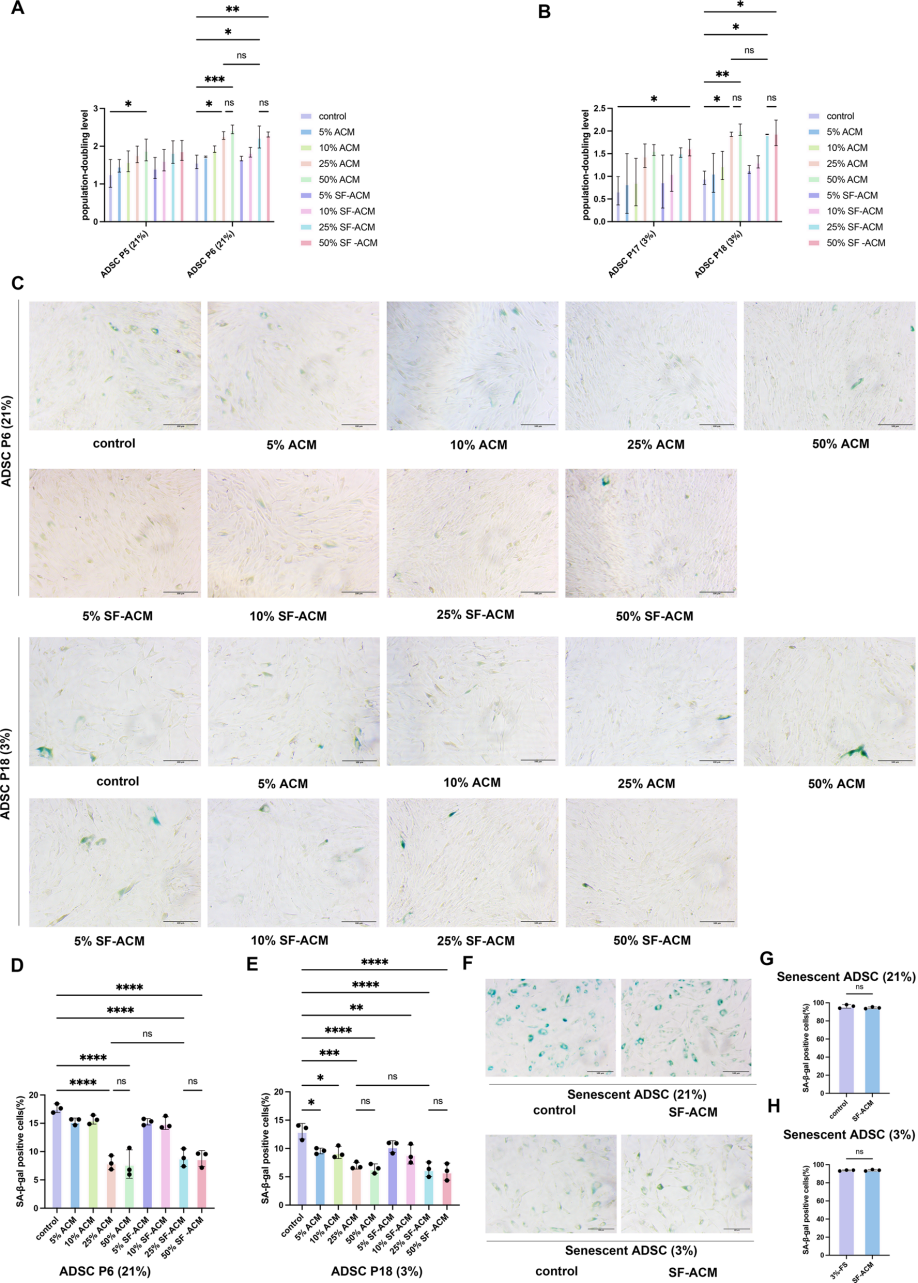
SF-ACM delays but does not reverse senescence in ADSCs. **A** and **B** Quantification of PDL in ADSC P6 (21%) and ADSC P18 (3%) after treatment with varying concentrations (5%, 10%, 25%, 50%) of ACM or SF-ACM. **C** Representative SA-β-gal staining images of ADSCs under different treatment conditions. **D** and** E** Quantification of the percentage of SA-β-gal–positive cells in ADSC P6 (21%) and ADSC P18 (3%). **F** Representative images of SA-β-gal staining in senescent cells following 25% SF-ACM treatment in fully senescent ADSCs derived from both models. **G** and** H** Quantification of the percentage of SA-β-gal–positive cells in fully senescent ADSCs derived from both models. Statistical significance was determined using unpaired two-tailed t tests. All data are presented as mean ± SD from three independent experiments. Statistical significance was assessed using one-way ANOVA followed by Tukey’s post hoc test unless otherwise noted; *P < 0.05, **P < 0.01, ***P < 0.001, ****P < 0.0001. Scale bar, 100 μm, applies to all images

We next assessed the functional efficacy of ACM and SF-ACM by applying them to ADSCs undergoing either oxidative stress–induced [ADSC P6 (21%)] or replicative [ADSC P18 (3%)] senescence. Treatment with both 25% and 50% concentrations significantly increased PDL and reduced SA-β-gal–positive cells in both models (Fig. 2A–E), with no significant difference between ACM and SF-ACM or between the two concentrations. Based on comparable efficacy and practicality, 25% SF-ACM was selected for subsequent experiments.

However, when applied to fully senescent ADSCs (SA-β-gal⁺ ~ 100%), SF-ACM failed to reverse senescence (Fig. 2F–H), indicating that SF-ACM primarily delays or prevents the progression of senescence rather than reversing established, irreversible senescent phenotypes.

### SF-ACM improves proliferation and migration in two ADSC senescence models

To evaluate the anti-senescence effects of SF-ACM, we assessed its impact on cell proliferation, cell cycle distribution, and migration capacity in two distinct senescence models: ADSC P6 (21%) and ADSC P18 (3%).

**Fig. 3:**
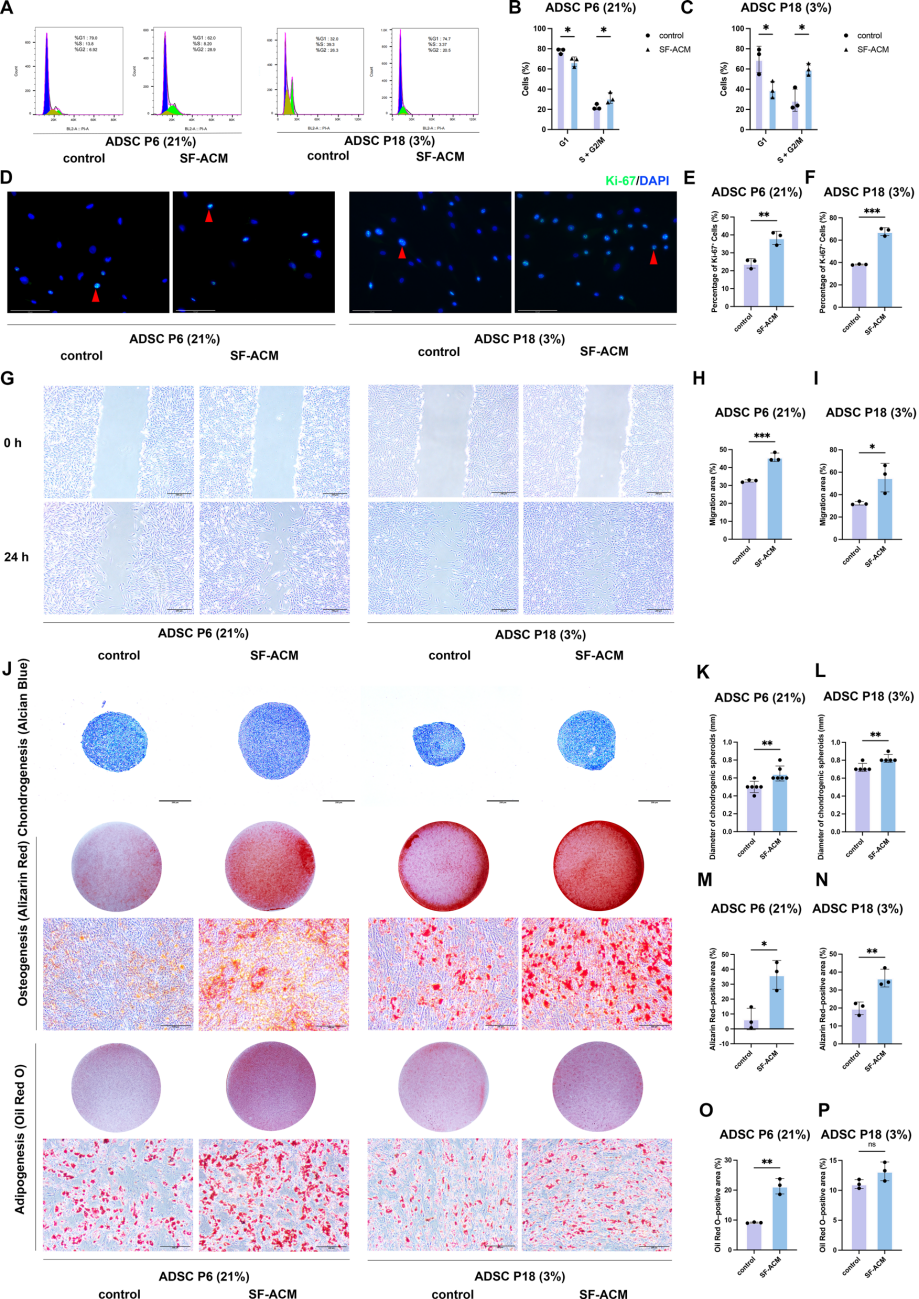
SF-ACM improves proliferation, migration, and multilineage differentiation in ADSC senescence models. **A**–**C** Flow cytometry analysis of cell cycle distribution in both models. Data were analyzed by two-way ANOVA. **D**–**F** Representative immunofluorescence staining of Ki-67 in both ADSC senescence models with or without SF-ACM treatment, with representative Ki-67–positive nuclei marked by red arrows, and quantification of the percentage of Ki-67–positive cells. Scale bar, 125 μm. **G**–**I** Representative scratch wound images in both models under different treatment conditions at 0 h and 24 h, and quantification of the percentage of migration area. Scale bar, 200 μm. **J** Representative images of multilineage differentiation following SF-ACM treatment. Chondrogenic differentiation was assessed by Alcian Blue staining; scale bar, 200 μm. Osteogenic (Alizarin Red S) and adipogenic (Oil Red O) differentiation were assessed by both bright-field microscopy and well-level images; scale bar, 100 μm (microscopy). **K** and **L** Quantification of chondrogenic differentiation by pellet diameter in both senescence models. n = 6 for ADSC P6 (21%); n = 5 for ADSC P18 (3%). **M** and **N** Quantification of the percentage of Alizarin Red S staining area in both models. **O** and **P** Quantification of the percentage of Oil Red O staining area in both models. All data are presented as mean ± SD from n = 3 unless otherwise indicated. Statistical significance was determined using unpaired two-tailed t tests unless otherwise noted. Statistical significance: *P < 0.05, **P < 0.01, ***P < 0.001, ****P < 0.0001

Flow cytometry analysis revealed that SF-ACM treatment significantly reduced the proportion of cells in the G1 phase and increased the proportion in the S and G2/M phases in both models (Fig. 3A–C), indicating enhanced progression through the cell cycle. Immunofluorescence staining showed a higher percentage of Ki67-positive cells in SF-ACM–treated cultures compared to untreated controls (Fig. 3D–F), consistent with increased proliferative activity.

Scratch wound assays further demonstrated that SF-ACM significantly promoted the migration capacity of both ADSC P6 (21%) and ADSC P18 (3%) (Fig. 3G–I). These results suggest that SF-ACM can partially restore functional properties such as proliferation and motility in ADSCs exhibiting distinct senescence phenotypes.

### SF-ACM enhances multilineage differentiation in two ADSC senescence models

To evaluate the effects of SF-ACM on the multilineage differentiation potential of ADSCs in two senescence models [ADSC P6 (21%) and ADSC P18 (3%)], adipogenic, osteogenic, and chondrogenic induction assays were performed.

Quantitative analysis of differentiation markers revealed that SF-ACM treatment significantly increased chondrogenic differentiation, as evidenced by a significant increase in pellet diameter after Alcian Blue staining in both ADSC P6 (21%) and ADSC P18 (3%) (Fig. 3J–L). Osteogenic differentiation, assessed by Alizarin Red S staining, was also significantly enhanced in both models (Fig. 3J and 3M–N). Adipogenic differentiation, indicated by Oil Red O staining, was significantly increased in ADSC P6 (21%) following SF-ACM treatment (Fig. 3J and O). Although adipogenic differentiation in ADSC P18 (3%) exhibited an upward trend following SF-ACM treatment, this change did not reach statistical significance (Fig. 3J and P).

These results indicate that SF-ACM enhances the differentiation capacity of ADSCs with distinct senescence phenotypes, particularly promoting osteogenic and chondrogenic lineages.

### SF-ACM modulates senescence-associated markers in two ADSC senescence models

To determine whether SF-ACM attenuates cellular senescence at the molecular level, we evaluated the expression of key senescence-associated markers in both the 21% O2–induced premature senescence model [ADSC P6 (21%)] and the replicative senescence model [ADSC P18 (3%)].

**Fig. 4:**
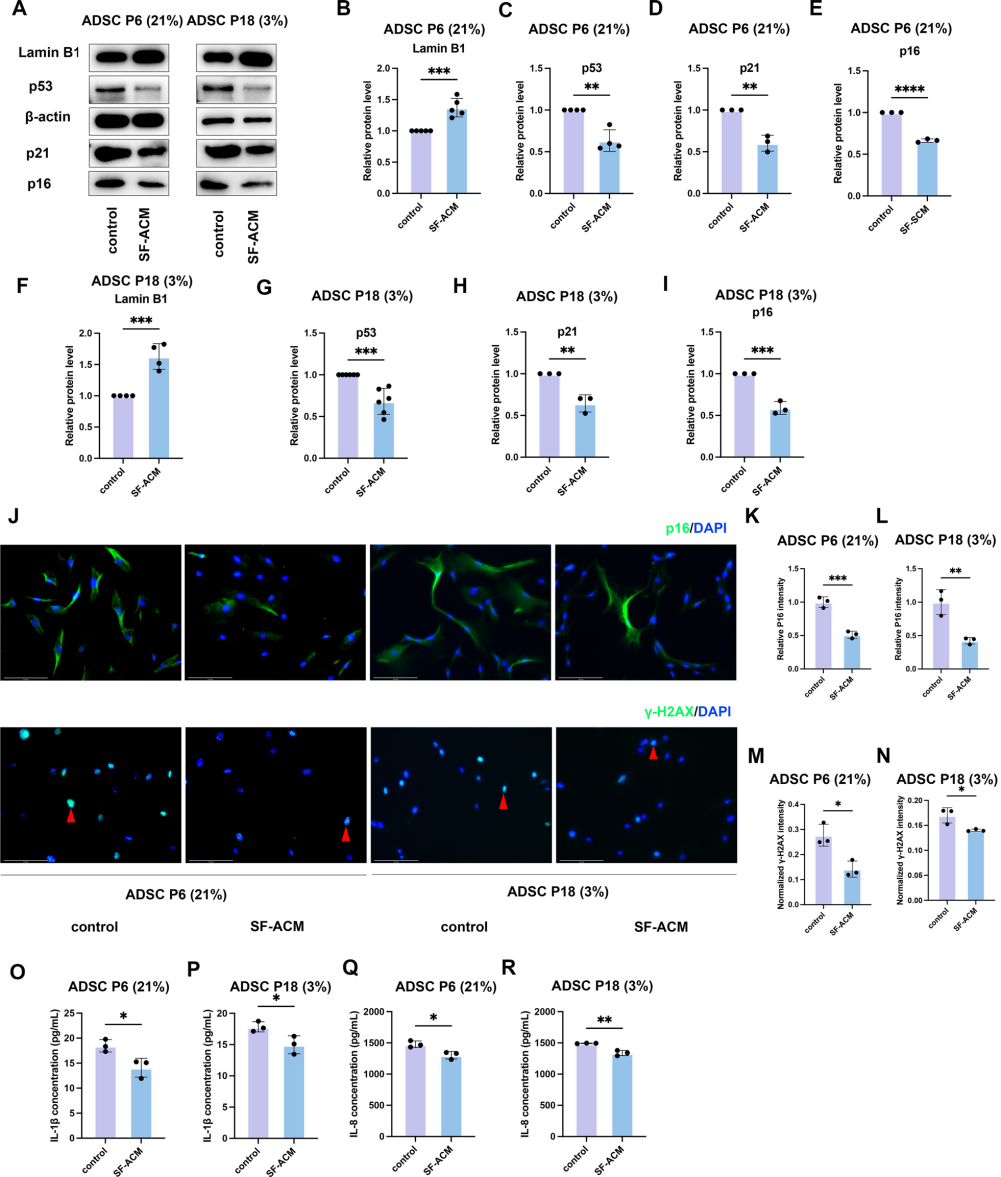
SF-ACM reduces senescence-associated markers in two ADSC senescence models. **A** Western blot analysis of p53, p21, p16, and Lamin B1 expression in ADSC P6 (21%) and ADSC P18 (3%) models with or without SF-ACM treatment. β-actin served as the loading control. **B**–**E** Densitometric quantification of p53, p21, p16, and Lamin B1 in ADSC P6 (21%), normalized to β-actin and expressed relative to untreated controls (set as 1.0). n = 4 (p53), n = 3 (p21), n = 3 (p16), n = 5 (Lamin B1). **F**–**I** Densitometric quantification of the same proteins in ADSC P18 (3%), normalized to β-actin and expressed relative to untreated controls. n = 6 (p53), n = 3 (p21), n = 3 (p16), n = 4 (Lamin B1). **J** Representative immunofluorescence images of p16 and γ-H2AX expression in both senescence models with or without SF-ACM treatment. Red arrows indicate representative γ-H2AX–positive nuclei. Scale bar, 125 μm. **K** and **L** Quantification of p16 signal intensities normalized to untreated controls. n = 3. **M** and **N** Quantification of relative γ-H2AX fluorescence intensity normalized to DAPI fluorescence. n = 3. **O**–**R** ELISA quantification of SASP factors IL-8 and IL-1β in culture supernatants from both models. n = 3. All data are presented as mean ± SD. Statistical significance was determined using unpaired two-tailed t tests. *P < 0.05, **P < 0.01, ***P < 0.001, ****P < 0.0001

Western blot analysis revealed that SF-ACM treatment significantly reduced protein levels of p53, p21, and p16, while increased Lamin B1 expression in both models (Fig. 4A). Densitometric quantification confirmed these changes (Fig. 4B–I). These markers, previously validated as indicators of senescence in both models, were significantly reversed by SF-ACM, indicating a rejuvenating effect at the molecular level. Immunofluorescence staining revealed a significant reduction in p16 and γ-H2AX signals after SF-ACM treatment (Fig. 4J–N). γ-H2AX is a phosphorylated histone variant, marks DNA double-strand breaks and signals activation of the DNA damage response. The reduction of these markers suggests that SF-ACM alleviates both cell cycle blockade and persistent DNA damage signaling in senescent ADSCs.

In addition, ELISA analysis demonstrated significantly decreased secretion of the SASP factors IL-8 and IL-1β in the culture supernatants following SF-ACM exposure (Fig. 4O–R). IL-8 and IL-1β are key proinflammatory cytokines that contribute to the SASP, reinforcing chronic inflammation and paracrine senescence in the tissue microenvironment.

Together, these findings indicate that SF-ACM alleviates hallmark features of cellular senescence in ADSCs, including dysregulated cell cycle control, nuclear envelope disintegration, genomic instability, and inflammatory secretome activation.

### SF-ACM alleviates senescence and fibrotic phenotypes in HDFs

To determine whether the anti-senescent effects of SF-ACM extend beyond ADSCs, we investigated its impact on HDFs, which play a crucial role in skin homeostasis and regeneration [25, 26]. HDFs are highly susceptible to replicative and oxidative stress-induced senescence, exhibiting distinct senescence-associated phenotypes [13, 27].

**Fig. 5:**
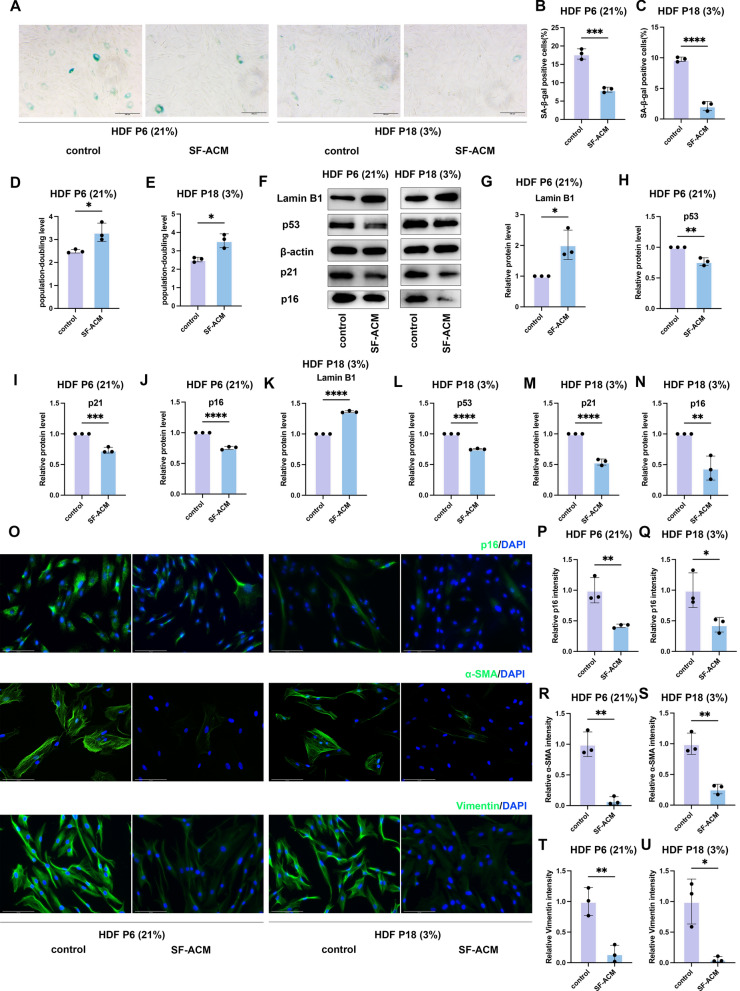
SF-ACM attenuates cellular senescence and fibrotic phenotypes in HDFs. **A** Representative images of SA-β-gal staining in HDF P6 (21%) and HDF P18 (3%) with or without SF-ACM treatment. Scale bars, 100 μm. **B** and **C** Quantification of the percentage of SA-β-gal–positive cells in HDF P6 (21%) and HDF P18 (3%), respectively. **D** and **E** Analysis of PDLs in both HDF models under different treatments. **F** Western blot of p53, p21, p16, and Lamin B1 in HDF P6 (21%) and HDF P18 (3%). **G**–**N** Quantification of p53, p21, p16, and Lamin B1 protein levels normalized to β-actin and expressed relative to untreated controls (set as 1.0). **O** Representative immunofluorescence images of p16, α-SMA, and Vimentin expression in HDF P6 (21%) and HDF P18 (3%). **S**cale bars, 125 μm. **P**–**U** Quantification of p16, α-SMA, and Vimentin signal intensities normalized to untreated controls. Data are presented as mean ± SD. n = 3 per group. P values were determined by unpaired two-tailed t test: *P < 0.05, **P < 0.01, ***P < 0.001, ****P < 0.0001

SF-ACM markedly reduced SA-β-gal positivity and increased PDL in both groups of HDFs—HDF P6 (21%) and HDF P18 (3%) (Fig. 5A–E)—indicating a significant anti-senescence effect. Western blot analysis revealed decreased expression of senescence markers p53, p21, and p16, along with increased Lamin B1 levels in both conditions (Fig. 5F–N). Immunofluorescence staining further showed a marked reduction in p16 expression following SF-ACM treatment (Fig. 5O–Q).

Because senescence is often accompanied by a fibrotic phenotype, we next assessed whether SF-ACM modulates fibrotic markers. Immunofluorescence results showed a significant reduction in α-smooth muscle actin (α-SMA) expression (Fig. 5O and R–S), indicating inhibition of fibroblast-to-myofibroblast transition (FMT). Moreover, SF-ACM decreased Vimentin expression (Fig. 5O and T–U), which may reflect reduced cytoskeletal remodeling, lower motility, or improved phenotypic stability.

Together, these results demonstrate that SF-ACM effectively reduces senescence-associated phenotypes and fibrotic markers in HDFs, suggesting cross-lineage efficacy and potential benefits in tissue remodeling and fibrosis regulation.

### SF-ACM is safe and improves aging skin and muscle

Our previous studies have shown that SF-ACM effectively suppresses senescence and preserves functional homeostasis in both ADSCs and HDFs in vitro. Given the fundamental role of cellular senescence in driving organismal aging, we hypothesized that SF-ACM may also exert anti-aging effects at the systemic level. To test this, we established two in vivo models—a D-galactose–induced progeroid model and a naturally aging model—to evaluate the potential of SF-ACM in modulating age-related tissue alterations.

**Fig. 6:**
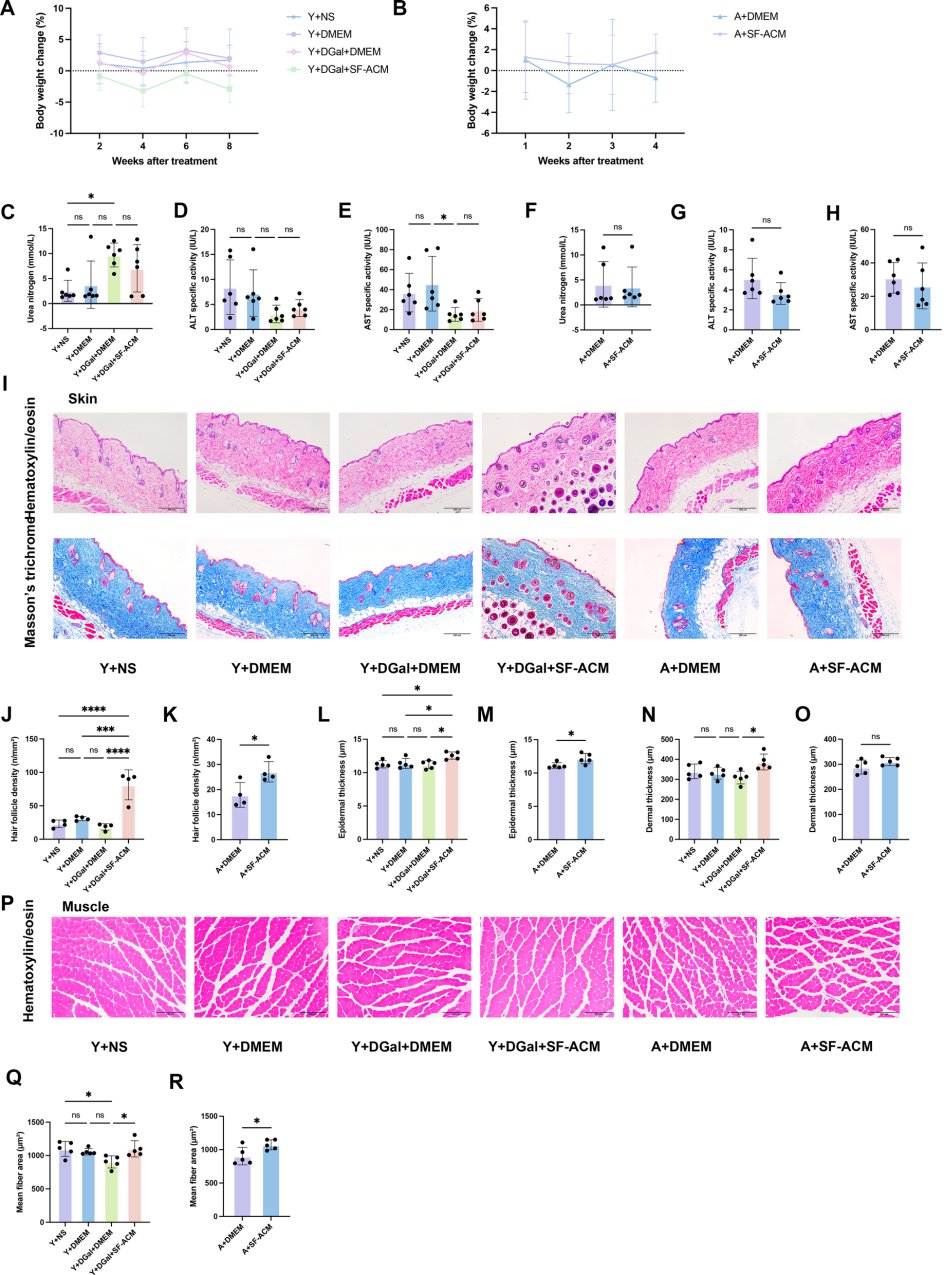
SF-ACM is safe in vivo and improves aging-related skin and muscle structure. **A** and **B** Percentage change in body weight during SF-ACM intervention in the D-galactose–induced progeroid model and the naturally aged model (n = 6). P values were determined by two-way ANOVA. **C**–**E** Serum levels of BUN (mmol/L), ALT (IU/L), and AST (IU/L) in the progeroid model (n = 6). **F**–**H** Same analyses in the naturally aged model (n = 6). **I** Representative images of H&E and Masson’s trichrome staining of dorsal skin from both models. Scale bar, 200 μm. **J** and **K** Quantification of hair follicle density (n/mm^2^) in the progeroid and naturally aged models (n = 4). **L** and **M** Quantification of epidermal thickness (μm) in the progeroid and naturally aged models (n = 5). **N** and **O** Quantification of dermal thickness (μm) in the progeroid and naturally aged models (n = 5). (**P**) Representative H&E images of gastrocnemius muscle cross-sections. Scale bar, 200 μm. **Q** and **R** Quantification of myofiber cross-sectional area (μm^2^) in the progeroid and aturally aged models. n = 5. All data are presented as mean ± SD. *P < 0.05, **P < 0.01, ***P < 0.001, ****P < 0.0001. Statistical significance was determined using an unpaired two-tailed t-test for the naturally aged models and one-way ANOVA for the progeroid models, unless otherwise specified. Y, young mice (4 months); A, aged mice (16 months); NS, normal saline; DGal, D-galactose

Given that SF-ACM does not reverse fully established senescence in vitro, 16-month-old mice were selected to model early-stage aging. Throughout the treatment period, all mice maintained normal feeding, sleep, and activity behaviors. The percentage change in body weight remained stable, with no significant differences among the four groups of 4-month-old mice (Fig. 6A) or between the two groups of 16-month-old mice (Fig. 6B), indicating that SF-ACM treatment was well tolerated in both young and aged mice. In addition, SF-ACM treatment did not significantly affect serum BUN, ALT, or AST levels in either model (Fig. 6C–H). These results indicate that SF-ACM is systemically well tolerated without inducing detectable hepatic or renal toxicity.

Histological analysis revealed that SF-ACM mitigated structural deterioration in skin and muscle. In both aging models, SF-ACM increased hair follicle density and epidermal thickness (Fig. 6I–M). In the progeroid model, multiple hair follicles notably entered the anagen (growth) phase (Fig. 6I). Dermal thickness was significantly improved in the progeroid group, with a similar trend in naturally aged mice (Fig. 6I, N–O). Both models showed thickened subcutaneous fat layers in controls, which appeared reduced after SF-ACM, although this was not quantitatively assessed (Fig. 6I).

In skeletal muscle, analysis of > 800 myofibers per sample showed a significant increase in cross-sectional area following SF-ACM treatment, indicating partial rescue of sarcopenia (Fig. 6P–R).

By contrast, SF-ACM had minimal effect on renal aging. Glomerular numbers were unchanged (Figure S2A–C), and Sirius Red staining revealed persistent interstitial fibrosis in both models, unaltered by treatment (Figure S2A and S2D–E).

Collectively, these findings indicate that SF-ACM is systemically well tolerated and ameliorates aging-associated changes in skin and muscle, especially when applied at early stages, although its impact on irreversible fibrotic changes such as renal fibrosis appears limited under current conditions.

### SF-ACM improves aging traits in skin, fat, and serum

**Fig. 7:**
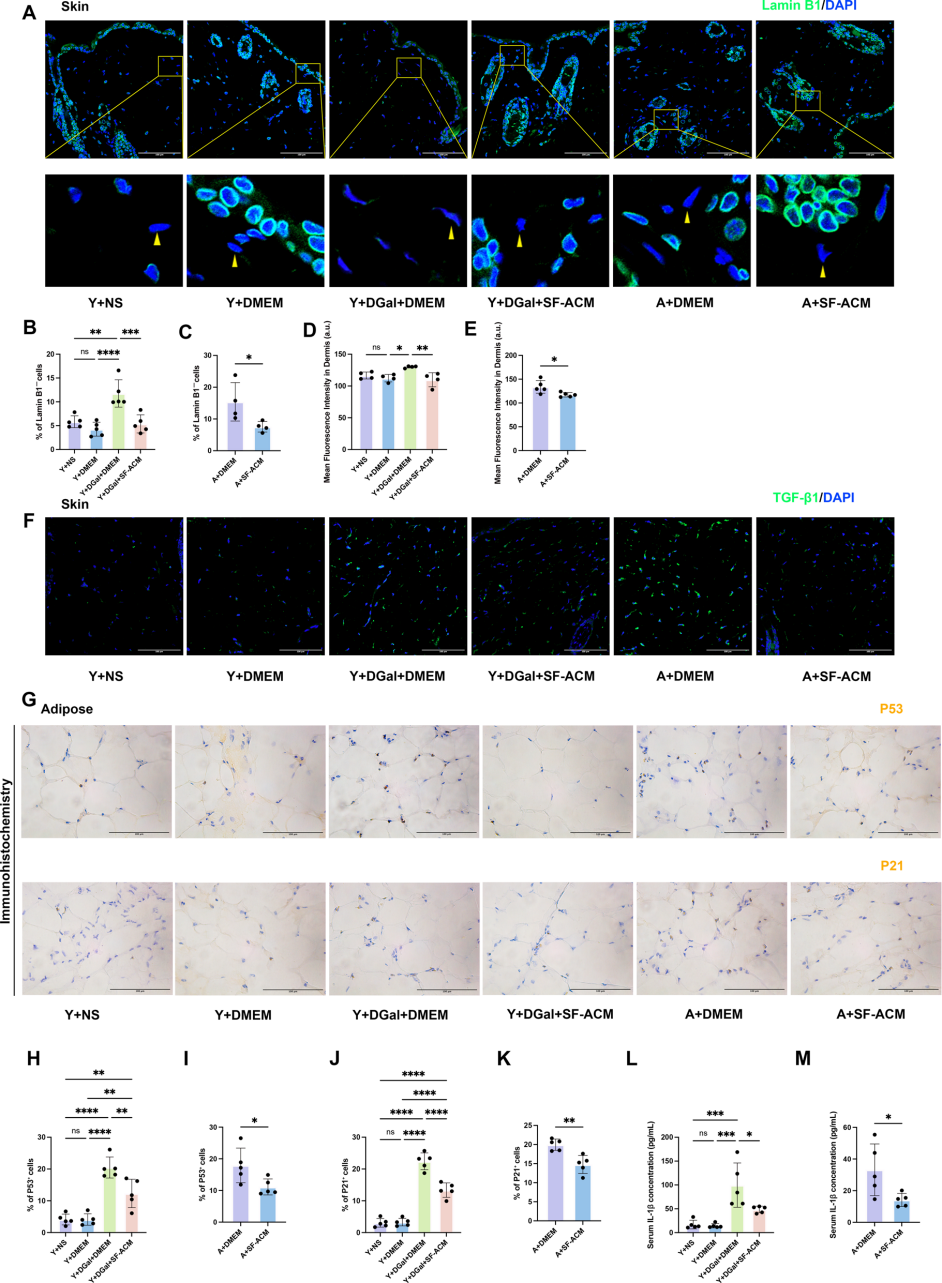
SF-ACM reduces aging-associated molecular features in skin and adipose tissue and suppresses systemic inflammation. **A** Representative immunofluorescence images of Lamin B1 in dorsal skin tissues from progeroid and naturally aged mice with or without SF-ACM treatment. Nuclei were counterstained with DAPI. Yellow arrows indicate Lamin B1–negative nuclei. Scale bar, 100 µm. **B** and **C** Quantification of the percentage of Lamin B1–deficient nuclei in skin from progeroid (**B**; n = 5) and naturally aged (**C**; n = 4) mice. **D** and **E** Quantification of dermal TGF-β1 mean fluorescence intensity (a.u.) in progeroid (n = 4) and naturally aged (n = 5) mice. **F** Representative immunofluorescence images of TGF-β1 expression in dorsal skin. Nuclei were counterstained with DAPI. Scale bar, 100 µm. **G** Representative immunohistochemical staining for p21 and p53 in inguinal adipose tissue. Scale bar, 100 µm. **H**–**K** Quantification of the percentage of p21⁺ and p53⁺ nuclei in adipose tissue from progeroid and naturally aged mice (n = 5 per group). **L** and **M** Serum IL-1β levels (pg/mL) measured by ELISA in progeroid and naturally aged mice (n = 5 per group). All data are presented as mean ± SD. *P < 0.05, **P < 0.01, ***P < 0.001, ****P < 0.0001. Statistical significance was determined using an unpaired two-tailed t test for the naturally aged models and one-way ANOVA for the progeroid models

To evaluate the in vivo anti-aging effects of SF-ACM, we assessed molecular features in skin and adipose tissues, along with serum inflammatory markers. In dorsal skin, Lamin B1–deficient nuclei—indicative of nuclear envelope disruption—were increased in D-galactose–treated mice but significantly reduced by SF-ACM in both aging models (Fig. 7A–C). TGF-β1 expression in the dermis was elevated in aging mice and significantly suppressed by SF-ACM treatment in both models (Fig. 7D–F).

In inguinal fat, immunohistochemistry revealed increased p21 and p53 expression in aging mice, which was significantly attenuated following SF-ACM administration (Fig. 7G–K), indicating reduced senescence signaling in adipose tissue.

SF-ACM also lowered systemic inflammation, as evidenced by reduced serum IL-1β levels in both models (Fig. 7L–M).

Together, these results demonstrate that SF-ACM alleviates key senescence markers in skin and fat while reducing circulating proinflammatory cytokines, highlighting its systemic anti-aging potential.

### IL-6 downregulation links SF-ACM to anti-senescence effects

**Fig. 8:**
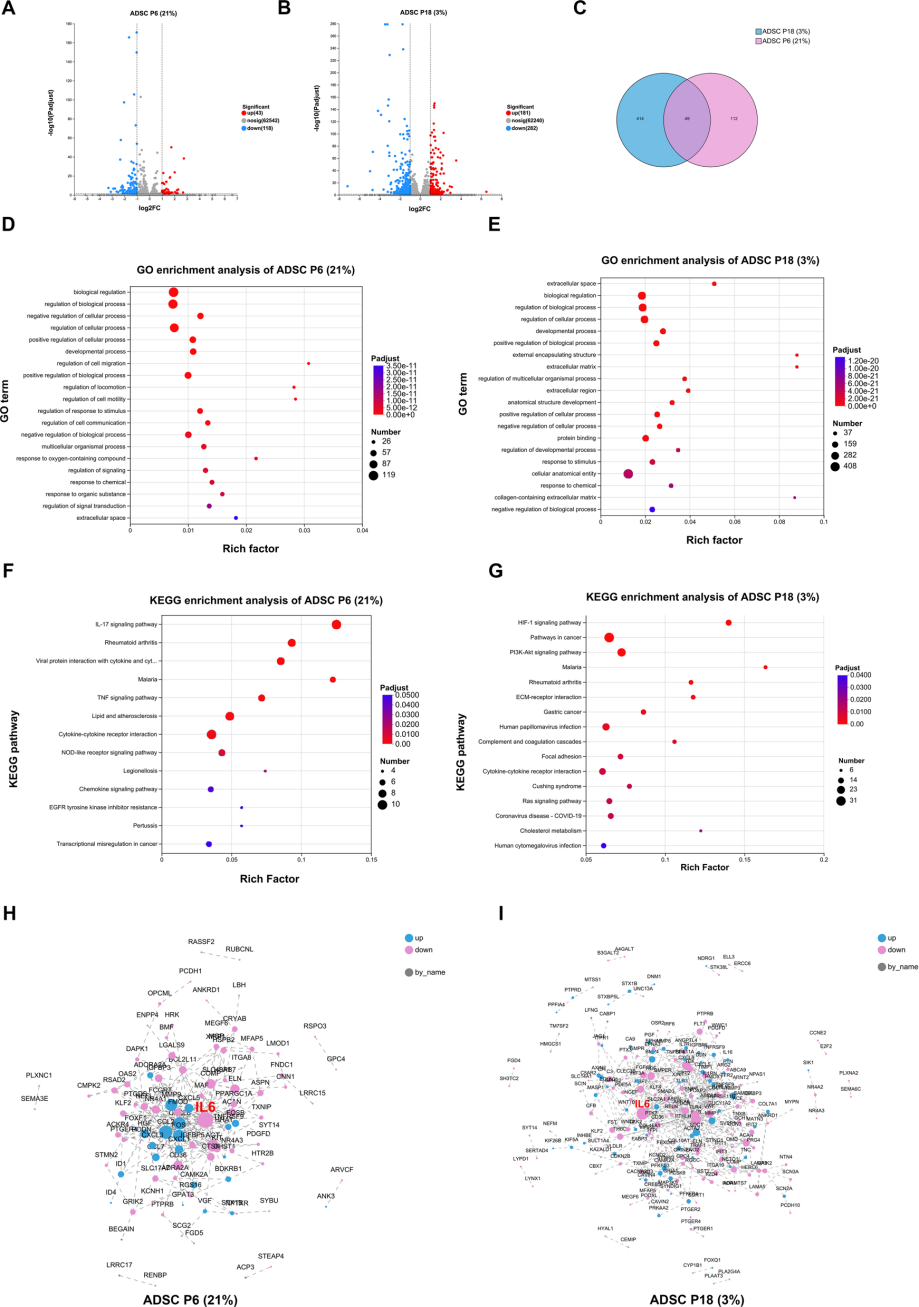
Transcriptomic profiling reveals IL-6 as a shared anti-aging target of SF-ACM. **A** and** B** Volcano plots showing DEGs in ADSC P6 (21%) and ADSC P18 (3%) following SF-ACM treatment. **C** Venn diagram showing the overlapping DEGs shared between the two models. **D** and **E** GO enrichment analysis of DEGs in ADSC P6 (21%) and ADSC P18 (3%) (top 20 terms; adjusted P < 0.05). **F** and** G** KEGG pathway enrichment analysis of DEGs in ADSC P6 (21%) and ADSC P18 (3%) (adjusted P < 0.05). **H** and **I** PPI networks of DEGs in ADSC P6 (21%) and ADSC P18 (3%). Data were analyzed using DESeq2 for differential expression (|log₂FC|≥ 1, adjusted P < 0.05). n = 3 biological replicates per group

To elucidate the molecular mechanisms underlying the anti-senescence effects of SF-ACM, we performed transcriptomic analysis of two senescence models of ADSCs: ADSC P6 (21%) and ADSC P18 (3%), both with or without SF-ACM treatment. In ADSC P6 (21%), 43 genes were upregulated and 118 were downregulated after SF-ACM exposure (Fig. 8A), while in ADSC P18 (3%), 181 genes were upregulated and 282 were downregulated (|log_2_FC|≥ 1, adjusted P < 0.05; Fig. 8B). A total of 49 differentially expressed genes (DEGs) were shared between the two models (Fig. 8C).

To interpret the functional significance of these DEGs, we conducted Gene Ontology (GO) enrichment analysis using an adjusted P value threshold of < 0.05. In ADSC P6 (21%), DEGs were primarily associated with biological regulation and stress response processes, including “regulation of response to stimulus” and “response to oxygen-containing compounds,” suggesting that SF-ACM may counteract oxidative stress–induced premature senescence by modulating intracellular signaling (Fig. 8D). In contrast, DEGs in ADSC P18 (3%) were enriched in extracellular matrix (ECM)–related terms such as “extracellular matrix,” “collagen-containing extracellular matrix,” and “external encapsulating structure,” implying that SF-ACM may mitigate replicative senescence by remodeling the ECM and altering the extracellular signaling environment (Fig. 8E).

To further examine the underlying pathways, KEGG enrichment analysis was performed. In ADSC P6 (21%), DEGs were significantly enriched in inflammatory signaling pathways, including the IL-17, TNF, and NOD-like receptor pathways, as well as cytokine–cytokine receptor interaction (Fig. 8F). In ADSC P18 (3%), SF-ACM–responsive genes were enriched in the HIF-1 signaling pathway, PI3K-Akt signaling, ECM–receptor interaction, focal adhesion, and pathways in cancer (Fig. 8G), suggesting regulatory roles in metabolic adaptation, adhesion, and cellular resilience to stress.

Despite distinct cellular contexts, three KEGG pathways—malaria, rheumatoid arthritis, and cytokine–cytokine receptor interaction—were commonly enriched in both senescence models (Fig. 8F–G). Among the 49 overlapping DEGs, *IL6*, *CXCL5*, *CXCL8*, and *TNFRSF9* were also mapped to these shared pathways. Notably, *IL6* was consistently downregulated and involved in all three, highlighting its potential role as a converging regulatory target of SF-ACM.

To explore its regulatory role, we constructed protein–protein interaction (PPI) networks. In both ADSC P6 (21%) and ADSC P18 (3%) DEG networks, *IL6* exhibited the highest degree of connectivity (Fig. 8H–I), identifying it as a central hub and potential key effector of SF-ACM’s action.

Collectively, these results suggest that SF-ACM modulates senescence via cell type–specific mechanisms involving inflammatory suppression and ECM remodeling. The consistent downregulation and central network positioning of *IL6* highlight its potential as a converging regulator mediating SF-ACM–induced rejuvenation across distinct aging phenotypes in ADSCs.

### SF-ACM inhibits IL-6/STAT3 to reduce ADSC senescence

Building upon transcriptomic findings that identified *IL6* as a key regulatory hub in SF-ACM–mediated anti-aging pathways, we further explored the relevance of this cytokine in cellular senescence. IL-6–mediated STAT3 activation is a well-established driver of inflammation-associated aging, contributing to both SASP maintenance and impaired regenerative capacity [28–30]. We therefore examined whether SF-ACM exerts its anti-senescent effects through inhibition of the IL-6/STAT3 signaling axis in two ADSC senescence models.

**Fig. 9:**
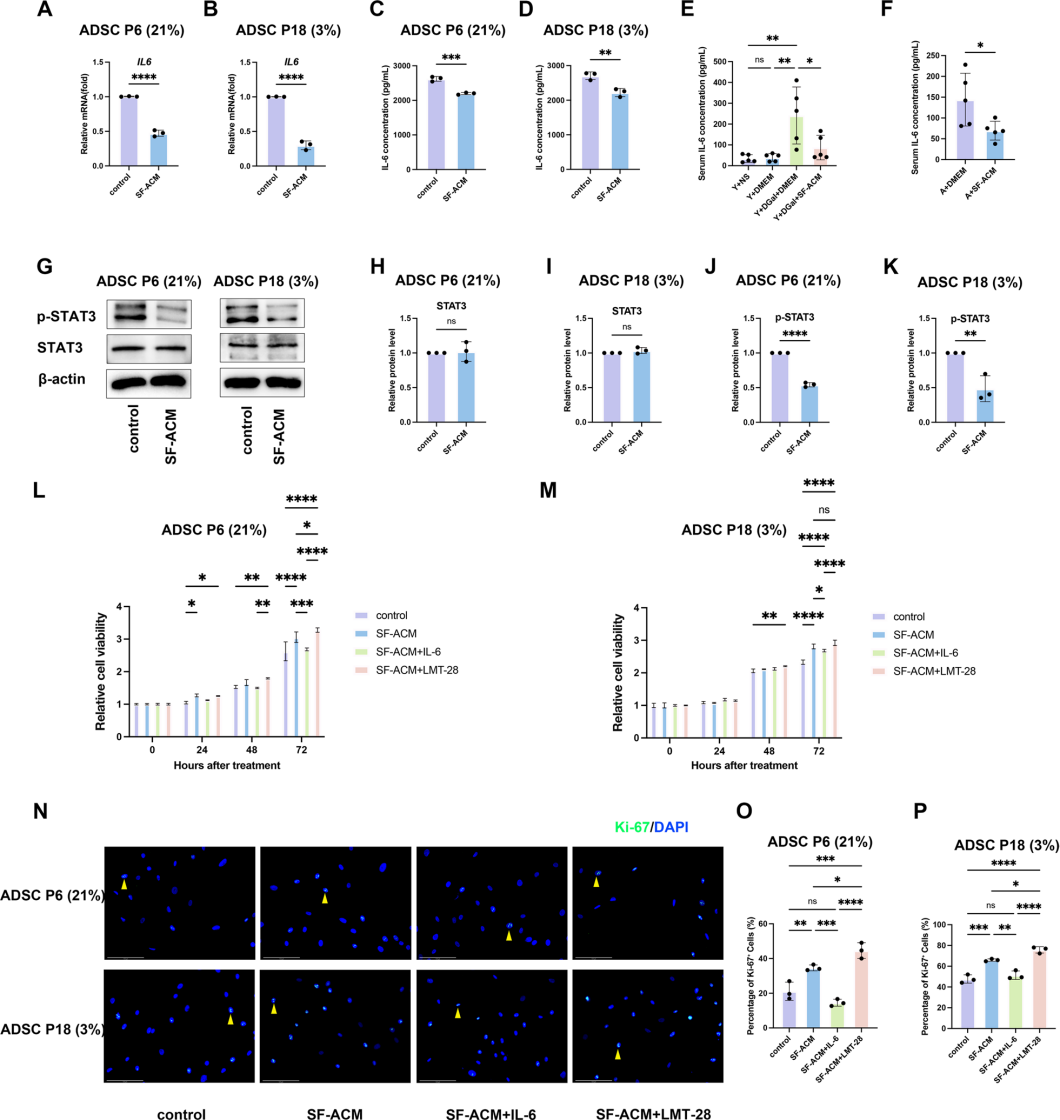
SF-ACM inhibits the IL-6/STAT3 pathway to alleviate senescence and promote proliferation in senescent ADSCs. **A** and **B** qPCR analysis of *IL6* mRNA expression, normalized to *ACTB* and expressed relative to untreated controls (set to 1.0). n = 3. **C** and **D** ELISA quantification of secreted IL-6 protein levels (pg/mL) in culture supernatants. n = 3. **E** and **F** Serum IL-6 concentrations (pg/mL) in progeroid and naturally aged mice with or without SF-ACM treatment. n = 5. **G** Representative Western blot images of total STAT3 and p-STAT3. **H**–**K** Densitometric quantification of total STAT3 and p-STAT3 protein levels, normalized to β-actin and expressed relative to untreated controls (set to 1.0). n = 3. **L** and **M** CCK-8 proliferation assays in ADSC P6 (21%) and ADSC P18 (3%). Data were normalized to the 0 h OD value of each group (set to 1.0). n = 3. **N** Representative immunofluorescence images of Ki-67–positive cells in ADSCs from each group. Nuclei were counterstained with DAPI. Yellow arrows indicate representative Ki-67⁺ nuclei. Scale bar, 125 μm. **O** and **P** Quantification of the percentage of Ki-67–positive cells in ADSC P6 (21%) and ADSC P18 (3%). n = 3. All data are presented as mean ± SD. *P < 0.05, **P < 0.01, ***P < 0.001, ****P < 0.0001. Statistical significance was assessed using unpaired two-tailed t test for two-group comparisons, one-way ANOVA for progeroid models, and two-way ANOVA for CCK-8 proliferation

qPCR and ELISA assays demonstrated that SF-ACM significantly reduced *IL6* mRNA expression and IL-6 protein secretion in both ADSC P6 (21%) and ADSC P18 (3%) (Fig. 9A–D). Correspondingly, serum IL-6 concentrations were significantly lower in SF-ACM–treated progeroid and naturally aged mice (Fig. 9E–F). Western blot analysis revealed that total STAT3 protein levels remained unchanged, whereas p-STAT3 levels were markedly decreased upon SF-ACM treatment (Fig. 9G–K), suggesting inhibition of IL-6–driven STAT3 activation.

To investigate functional consequences, ADSCs were treated with SF-ACM alone, or in combination with recombinant IL-6 or LMT-28, a selective inhibitor of the IL-6/STAT3 signaling pathway. CCK-8 assays showed that IL-6 partially reversed the proliferative effect of SF-ACM, while LMT-28 further enhanced proliferation, though without statistical significance in the P18 model (Fig. 9L–M). Similarly, immunofluorescence staining for Ki-67 indicated that SF-ACM increased the number of Ki-67⁺ cells, an effect reversed by IL-6 and further enhanced by LMT-28 (Fig. 9N–P), supporting the role of STAT3 suppression in SF-ACM–induced proliferation.

**Fig. 10:**
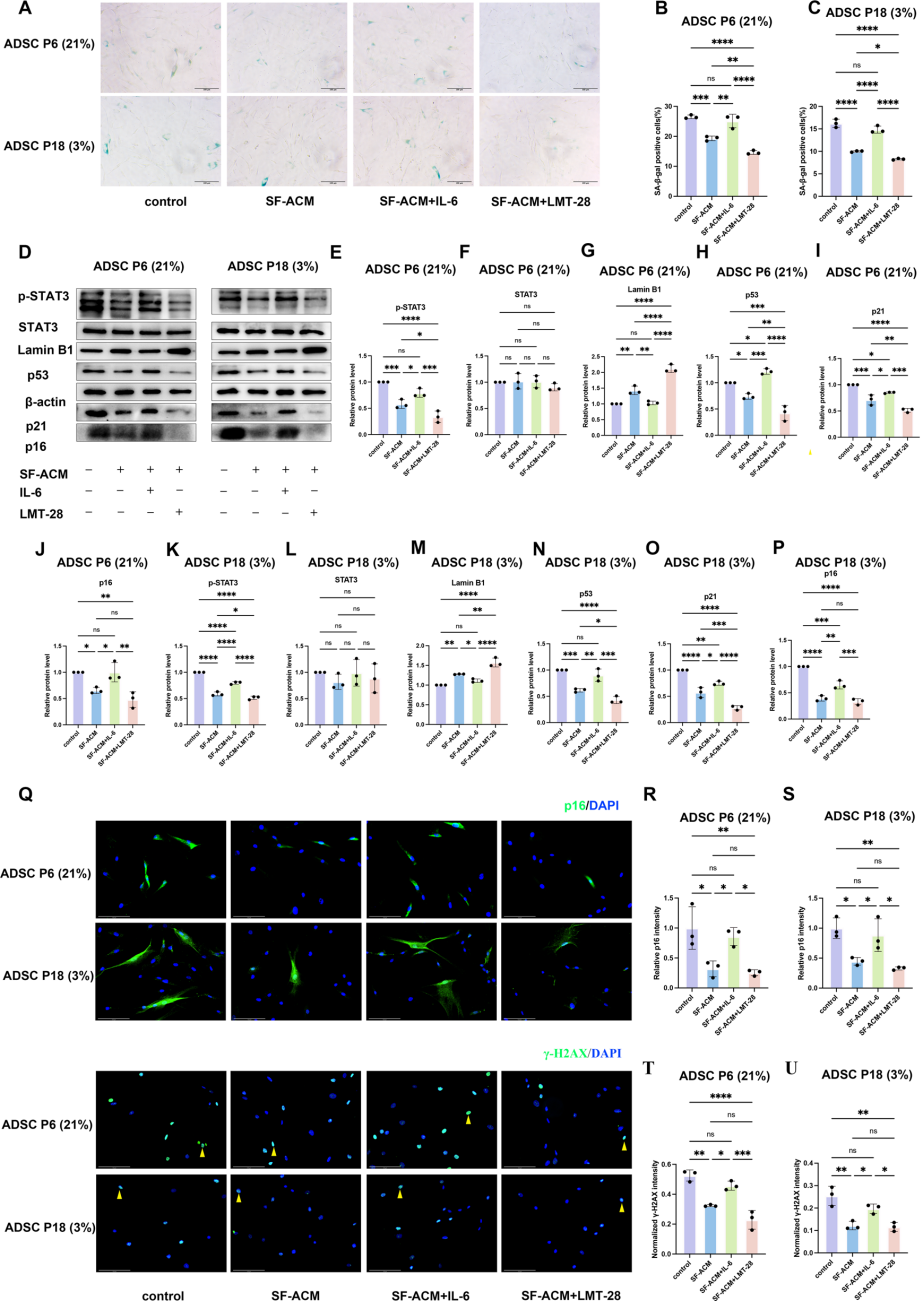
IL-6/STAT3 pathway modulation alters senescence marker expression in ADSCs. **A** Representative images of SA-β-gal staining in ADSCs under different treatment conditions. Scale bar, 100 μm. **B** and **C** Quantification of the percentage of SA-β-gal–positive cells in ADSC P6 (21%) and ADSC P18 (3%) (n = 3). **D** Representative Western blots showing expression of p53, p21, p16, Lamin B1, STAT3 and p-STAT3 in ADSCs treated with SF-ACM, IL-6, or LMT-28. **E**–**P** Quantification of p53, p21, p16, Lamin B1, STAT3, and p-STAT3 protein levels, normalized to β-actin (n = 3). **Q** Representative immunofluorescence images of p16 and γ-H2AX staining in ADSC P6 (21%) and ADSC P18 (3%). Nuclei were counterstained with DAPI. Yellow arrows indicate representative γ-H2AX⁺ nuclei. Scale bar, 125 μm. **R** and** S** Quantification of p16 signal intensities normalized to untreated controls (n = 3). **T** and **U** Quantification of the percentage of γ-H2AX–positive cells (n = 3). All data are presented as mean ± SD. *P < 0.05, **P < 0.01, ***P < 0.001, ****P < 0.0001. Statistical significance was determined by one-way ANOVA followed by Tukey’s post hoc test

Furthermore, SA-β-gal staining showed that IL-6 attenuated the SF-ACM–mediated reduction in senescent (SA-β-gal⁺) cell numbers, whereas LMT-28 amplified this reduction in both models (Fig. 10A–C). Western blot analysis confirmed that IL-6 addition upregulated senescence markers p21, p16, and p53 and decreased Lamin B1 expression, along with elevated p-STAT3 levels. In contrast, LMT-28 treatment had opposite effects (Fig. 10D–P). Immunofluorescence analysis further supported these results: IL-6 increased p16 expression and γ-H2AX nuclear foci, whereas LMT-28 showed a reduction trend, although not statistically significant in all conditions (Fig. 10Q–U).

Collectively, these results demonstrate that SF-ACM alleviates ADSC senescence and promotes proliferation by downregulating IL-6 expression, inhibiting STAT3 phosphorylation, and suppressing downstream senescence-associated pathways.

## Discussion

Explant cultures are known to mimic the early activation state of tissues following injury, which may underlie the regenerative and anti-aging potential of ACM [31, 32]. In our study, we observed that SF-ACM exhibited anti-senescent efficacy comparable to that of ACM. These findings indicate that, even in the absence of serum, explanted adipose tissue can adopt an intrinsic activation state capable of releasing bioactive secretomes.

SF-ACM attenuated senescence in both oxidative and replicative ADSC models in vitro and in aged mouse adipose tissue in vivo. Its effects were also observed in mesenchymal-rich tissues such as skin and muscle, raising the possibility that SF-ACM exerts broader tissue-level benefits. While it remains uncertain whether SF-ACM primarily targets senescent cells or preserves stem cell pools, these findings highlight its potential relevance to tissue rejuvenation strategies.

As ADSCs are widely studied for their immunomodulatory and regenerative functions, maintaining their proliferative and differentiation competence is essential for therapeutic use [11]. Here, we found that SF-ACM not only delayed senescence but also enhanced ADSC migration and osteo/chondrogenic differentiation. These improvements in cellular functionality may enhance the quality of ADSC-derived products, such as conditioned media or extracellular vesicles (EVs), and could increase their potential therapeutic relevance [33, 34].

Mechanistically, SF-ACM mitigated ADSC senescence through distinct but converging pathways in oxidative stress–induced [ADSC P6 (21%)] and replicative [ADSC P18 (3%)] senescence models. Transcriptomic analyses revealed differential regulation of stress response and extracellular matrix pathways, both converging on IL-6 downregulation—a central SASP component. Protein interaction and functional assays further identified IL-6 as a key regulatory hub. Exogenous IL-6 supplementation significantly reversed the anti-senescent effects of SF-ACM, as shown by increased expression of senescence-associated markers (p21, p16, p53), elevated SA-β-gal activity, and reactivation of IL-6/STAT3 signaling (p-STAT3), suggesting that IL-6/STAT3 signaling functions upstream in driving senescence. Consistently, treatment with SF-ACM combined with LMT-28, a small-molecule STAT3 inhibitor, further enhanced the anti-senescent effects, reinforcing the causal role of IL-6/STAT3 inhibition. Collectively, these data support a senomorphic role for SF-ACM in modulating the IL-6/STAT3 axis and reshaping the inflammatory microenvironment to counteract age-associated cellular dysfunction [28, 29, 35].

In recent years, increasing attention has been paid to the limitations of serum-based culture systems, including the risk of xenogeneic contamination, batch variability, and potential immunogenicity when applied to therapeutic products [23, 24]. To overcome these drawbacks, serum-free culture systems have been increasingly developed, such as STK, a defined serum-free formulation suitable for MSC culture, which has been shown to enhance MSC immunosuppressive and improve their therapeutic potential [36, 37]. In parallel, SF-ACM is also generated entirely under serum-free conditions, providing an alternative strategy that harnesses the intrinsic secretory activity of tissue explants. Rather than representing a passive culture by-product, SF-ACM likely reflects an activated secretome induced by adhesion, stress, and tissue-intrinsic repair cues. Unlike single-molecule or narrowly targeted agents, its multi-target activity may enable broader modulation of senescence-related phenotypes. Moreover, SF-ACM retains measurable bioactivity after long-term storage at –80 °C, facilitating scalable production and distribution [38, 39]. It is generated through a simple explant culture approach without enzymatic digestion or complex purification, making it technically accessible and potentially easier to standardize. The consistent effects observed across mesenchymal cell types, together with its defined mechanism of action and stability, suggest that SF-ACM could serve as a broadly applicable and potentially scalable senotherapeutic approach for fibrotic, inflammatory, and degenerative conditions [38–41].

Nonetheless, this study has several limitations. First, although both ACM and SF-ACM suppress ADSC senescence in vitro, we did not perform in vivo experiments with ACM due to xenogeneic serum limitations and translational considerations; therefore, future studies will need to evaluate their comparative efficacy in vivo. Second, the active components of SF-ACM remain undefined, and contributions from proteins, EVs, lipids, and metabolites require further investigation [42, 43]. Third, functional outcomes such as barrier restoration, strength, or lifespan were not evaluated and should be addressed in future studies. Moreover, intraperitoneal delivery may not be clinically optimal, and alternate delivery methods should be explored. Finally, donor variability and culture conditions may affect potency, suggesting that future work should investigate optimal donor selection and culture parameters [12].

## Conclusion

This study suggests that SF-ACM is a reproducible senotherapeutic candidate that attenuates cellular senescence in ADSCs and HDFs, preserves ADSC differentiation potential, and ameliorates aging-associated tissue changes in mice. Mechanistically, its effects are associated with modulation of the IL-6/STAT3 signaling pathway. Its xenogeneic-free composition, relative ease of production, and observed efficacy support further investigation of SF-ACM as a potential intervention for aging-related and degenerative conditions.

## Supplementary Information

Additional file 1.

## Data Availability

The datasets used and/or analysed during the current study are available from the corresponding author on reasonable request.

## Abbreviations

SASP: Senescence-associated secretory phenotype
MSCs: Mesenchymal stem cells
ADSCs: Adipose-derived mesenchymal stem cells
DMEM: Dulbecco’s Modified Eagle Medium
SF-ACM: Serum-free adipose-conditioned medium
HDFs: Human dermal fibroblasts
ADSC P6 (3%): Passage 6 ADSCs cultured at 3% O_2_
ADSC P6 (21%): Passage 6 ADSCs cultured at 21% O_2_
ADSC P18 (3%): Passage 18 ADSCs cultured at 3% O_2_
PDL: Population doubling level
FBS: Fetal bovine serum
ACM: Adipose-conditioned medium
α-SMA: α-Smooth muscle actin
FMT: Fibroblast-to-myofibroblast transition
BUN: Blood urea nitrogen
ALT: Alanine aminotransferase
AST: Aspartate aminotransferase
DEGs: Differentially expressed genes
GO: Gene ontology
ECM: Extracellular matrix
PPI: Protein–protein interaction
qPCR: Quantitative PCR
p-STAT3: Phosphorylated STAT3
EVs: Extracellular vesicles
NS: Normal saline
PBS: Phosphate-buffered saline
OD: Optical density
TPM: Transcripts per million
H&E: Hematoxylin and eosin
BSA: Bovine serum albumin
SD: Standard deviation
