# Supplementary material for: A serum-free adipose-conditioned medium delays stem cell senescence and maintains tissue homeostasis via IL-6/STAT3 axis suppression
Source: Stem Cell Res Ther. 2025 Nov 28;16:668. doi: 10.1186/s13287-025-04721-8 (PMC12664241; doi:10.1186/s13287-025-04721-8)
Supplement: Supplementary file 1 — Additional file 1. [file 13287_2025_4721_MOESM1_ESM.docx]

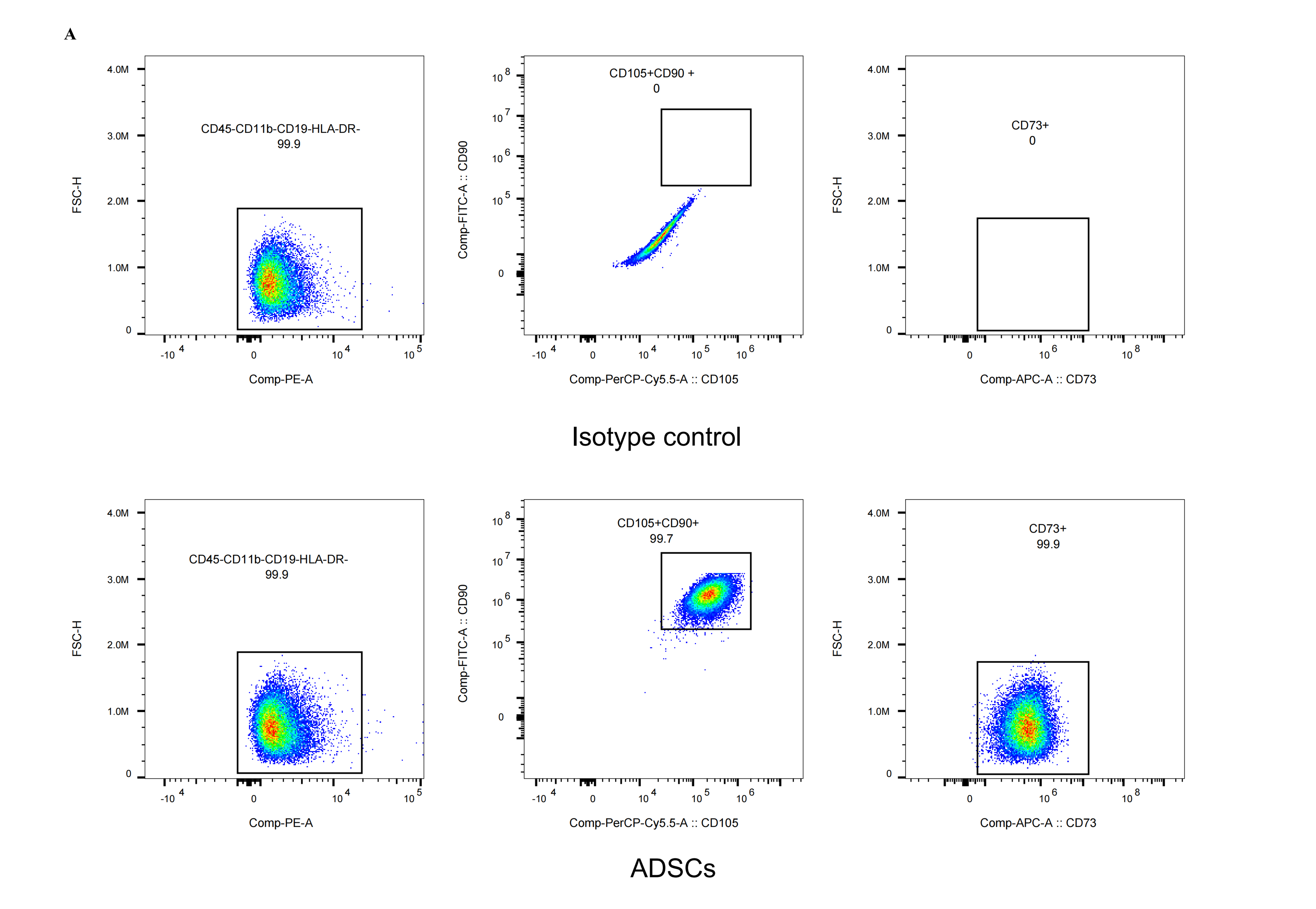
Figure S1. Characterization of primary human adipose-derived mesenchymal stem cells (ADSCs) by flow cytometry. The upper row shows isotype control histograms. The lower row shows staining results for positive markers (CD105, CD90, and CD73) and negative markers (CD45, CD11b, CD19, and HLA-DR).


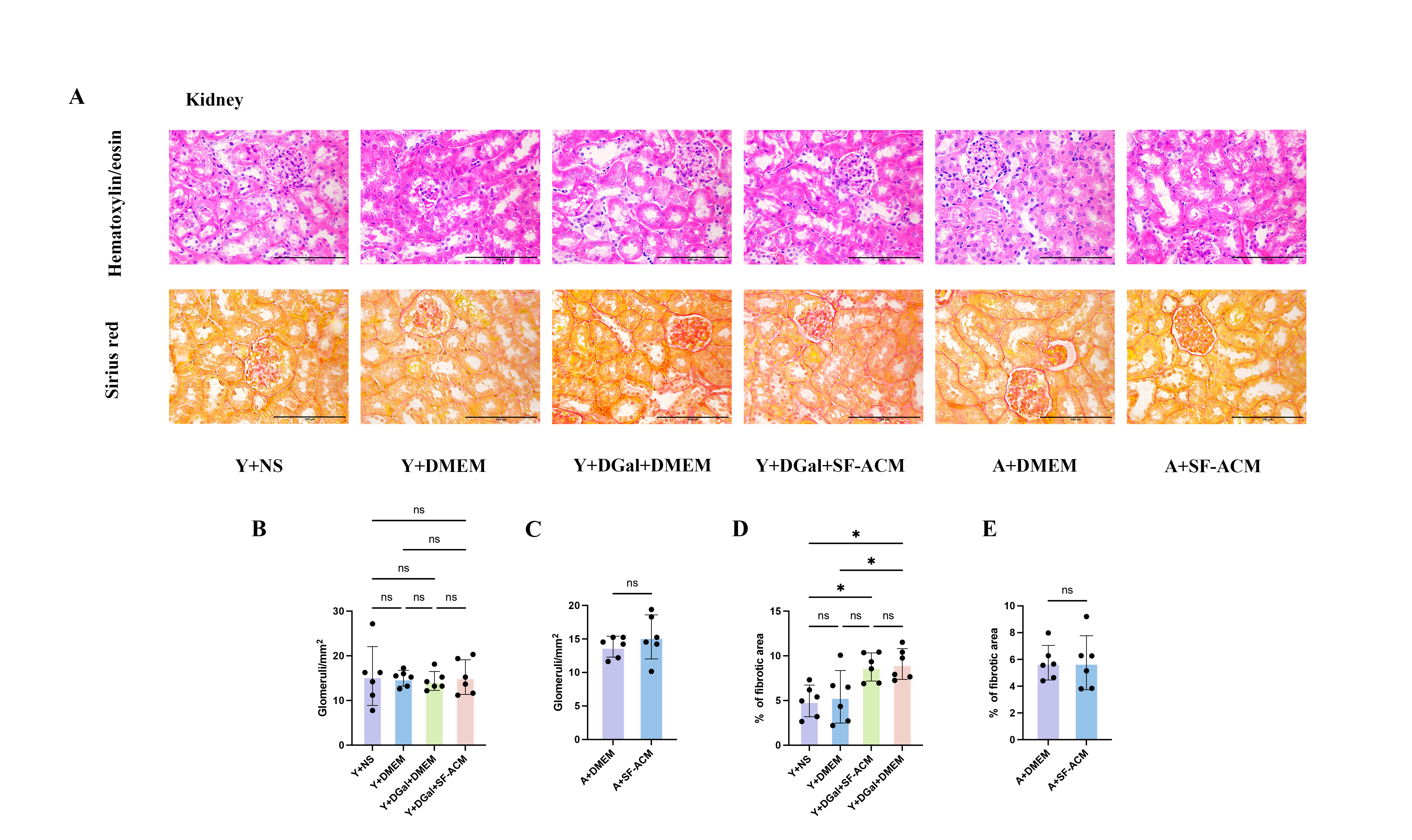
Figure S2. Histological analysis of renal structures in D-galactose–induced and naturally aged mice with or without SF-ACM treatment. (**A**) Representative hematoxylin-eosin and sirius red staining images of the kidney cortex. Scale bars, 100 μm. (**B** and **C**) Quantification of glomerular number in the D-galactose model and natural aging model. n = 5. (**D** and **E**) Assessment of interstitial fibrosis reveals severe fibrotic deposition in both aging models. n = 5. All data are presented as mean ± SD (n = 4 per group). Scale bars, 100 μm. *P < 0.05, **P < 0.01, ***P < 0.001, ****P < 0.0001. Statistical significance was determined by one-way ANOVA followed by Tukey’s post hoc test.
